# Investigation of the Semiconductor Properties and Chronoamperometry of BI_2_SE_3_ Thin Films Obtained by Electrochemical Deposition

**DOI:** 10.3390/nano16160991

**Published:** 2026-08-11

**Authors:** Sevinj P. Javadova, Vusala A. Majidzade, Ibrahim Kasumogly, Nahida N. Musayeva, Nadir A. Abdullayev, Samira F. Jafarova, Elvin J. Ahmadov, Dunya M. Babanly, Asmat N. Azizova, Akif Sh. Aliyev

**Affiliations:** 1 “Institute of Chemistry” PLE Ministry of Science and Education of the Republic of Azerbaijan, AZ 1173 Baku, Azerbaijan; sevinc92.mva@gmail.com (S.P.J.); ceferova.samira@list.ru (S.F.J.); elvin.j.ahmadov@gmail.com (E.J.A.); akifaliyev55@mail.ru (A.S.A.); 2 “Institute of Physics” PLE Ministry of Science and Education of the Republic of Azerbaijan, AZ 1173 Baku, Azerbaijan; iibrahimqasimoqlu@gmail.com (I.K.); nmusayeva@physics.science.az (N.N.M.); abnadir@mail.ru (N.A.A.); 3 Chemistry Department, Azerbaijan State Oil and Industry University (ASOIU), 34 Azadliq Ave., AZ 1010 Baku, Azerbaijan; dunya.babanly@ufaz.az; 4 Chemistry Department, French-Azerbaijani University (UFAZ), 183 Nizami Str., AZ 1000 Baku, Azerbaijan; 5 Azerbaijan Medical University Scientific Research Centre, 163A Samad Vurgun, Nasimi District, AZ 1022 Baku, Azerbaijan; esmet@rambler.ru

**Keywords:** Bi_2_Se_3_ thin films, electrodeposition, semiconductor properties, infrared photoresponse, chronoamperometry, Raman spectroscopy

## Abstract

Thin Bi_2_Se_3_ films were successfully electrodeposited from a non-aqueous ethylene glycol-based electrolyte and subsequently underwent thermal treatment in an Ar atmosphere to improve crystallinity. The phase and compositional properties of the films were characterized by X-ray diffraction (XRD), scanning electron microscopy (SEM), energy-dispersive X-ray spectroscopy (EDS), Fourier-transform infrared spectroscopy (FTIR), and Raman spectroscopy. XRD and Raman analyses confirmed the formation of crystalline rhombohedral Bi_2_Se_3_, while FTIR measurements indicated minor surface oxidation. The electrical and semiconductor properties of the films were investigated through current–voltage and temperature-dependent conductivity measurements. The activation energy associated with intrinsic conduction was determined to be 0.25 eV, while the temperature sensitivity coefficient and temperature coefficient of resistance were found to be 2903 K and 0.032–0.013 K^−1^, respectively. Infrared photoconductivity measurements in the wavelength range of 2800–4000 nm revealed a pronounced photoresponse near the band-gap energy of Bi_2_Se_3_. The obtained results demonstrate the potential of electrodeposited Bi_2_Se_3_ thin films for infrared-sensitive semiconductor and optoelectronic applications.

## 1. Introduction

Currently, with the advancement of technology, the demand for multifunctional new materials based on metal chalcogenides is growing rapidly [1,2]. These materials are typically used in the form of thin films [3,4,5].

One such interesting functional material is Bi_2_Se_3_ [6,7,8]. Bi_2_Se_3_ is a promising n-type semiconductor material belonging to the V^B^–VI^A^ group, with a band gap of 0.34 eV in monocrystalline form and 0.9–2.3 eV in thin-film form [9,10,11,12]. Photoemission studies of bismuth selenide have shown that it is a three-dimensional topological insulator with non-degenerate spin states, which makes it an ideal candidate for applications in electronics and spintronics [13,14,15,16,17,18]. Bi_2_Se_3_ is a compound with a rhombohedral crystal structure, where the unit cell can be schematically represented by three quintuple layers stacked along the [001] crystallographic direction, with lattice parameters of *a* = *b* = 0.416 nm and *c* = 2.861 nm. Each layer of bismuth selenide consists of five atomic sublayers arranged in the sequence Se–Bi–Se–Bi–Se [12,19,20,21]. These layers are held together by weak van der Waals interactions [20,21,22]. This unique anisotropic layered crystal structure enhances the electrical conductivity of bismuth selenide [23].

Up to now, various deposition methods have been employed for the synthesis of bismuth selenide thin films, including thermal evaporation [24,25,26], chemical vapor deposition (CVD) [21,27], chemical bath deposition (CBD) [28,29], pulsed laser ablation [30], electrodeposition [6,7,8,31,32], hydrothermal synthesis [15,33], pulsed magnetron sputtering [14], molecular beam epitaxy (MBE) [34,35], and other techniques.

Among these synthesis methods, electrochemical deposition is relatively simple and highly cost-effective. It does not require expensive equipment, including vacuum systems, and enables thin-film growth at relatively low temperatures. Although post-deposition thermal treatment may be required to improve crystallinity, the overall thermal budget remains lower than that of many conventional thin-film fabrication techniques (compared with conventional heat treatment, annealing generally requires more stringent processing conditions, including the use of a vacuum environment and relatively high temperatures) [36,37]. Moreover, the method is environmentally friendly and highly selective [38,39].

Recent studies have identified morphology control of Bi_2_Se_3_ materials as one of the major directions of innovation [40,41]. Bi_2_Se_3_ nanostructures synthesized in the form of nanoflowers, nanoplates, nanowires, and quantum dots exhibit a high surface area and excellent charge carrier mobility, resulting in enhanced performance in photocatalysis, gas sensing, and thermoelectric applications [42,43,44,45,46]. Heterostructures fabricated from low-dimensional, 2D, and 3D materials serve as a platform that can be used for many different purposes and enable novel device functionalities. However, their fabrication is still challenging due to issues such as interfacial contamination, defect formation, and limitations in large-scale production [47]. In this context, Bi_2_Se_3_-based heterostructures have attracted considerable attention [48]. In particular, composite structures formed with WSe_2_, TiO_2_, and ZnO demonstrate significantly improved optical and photoelectrochemical properties [49,50,51].

In recent years, the application of Bi_2_Se_3_ material in diode- and heterojunction-based electronic devices has attracted great interest and extensive research is being conducted in the direction of preparing Bi_2_Se_3_-based diode structures [52,53,54,55]. Among them, Si/Bi_2_Se_3_ heterojunctions are one of the most extensively studied systems, exhibiting low operating voltage, high photosensitivity, and excellent rectification characteristics [56]. In addition, ZnO/Bi_2_Se_3_, TiO_2_/Bi_2_Se_3_, and MoSe_2_/Bi_2_Se_3_ heterostructures are widely employed in modern optoelectronic devices because of their high photocurrent response and efficient charge carrier transport [57,58,59].

Bi_2_Se_3_ diodes based on nanoplate and nanowire morphologies provide a larger active surface area and faster charge transport pathways, which significantly enhance the sensitivity of infrared photodetectors [60]. Moreover, Bi_2_Se_3_ diodes fabricated on flexible substrates are considered highly promising for applications in flexible electronics and wearable sensor technologies [61].

The aim of the present study is to investigate the semiconductor properties, photoconductivity behavior, and nucleation mechanisms of electrodeposited Bi_2_Se_3_ thin films employed in diode structures.

While electrodeposition of Bi_2_Se_3_ thin films has been previously reported, comprehensive studies simultaneously addressing their phase characteristics, semiconductor behavior, far-infrared photoconductivity, and nucleation mechanisms remain limited. Therefore, the novelty of the present work is not only to obtain Bi_2_Se_3_ thin films by electrodeposition (from the electrolyte (M): 0.07 Bi(NO_3_)_3_ × 5H_2_O + 0.03 H_2_SeO_3_ + C_6_H_8_O_7_) but also to establish growth mechanisms, correlations between their electrical properties, and photoresponse characteristics.

## 2. Materials and Methods

**Figure 1:**
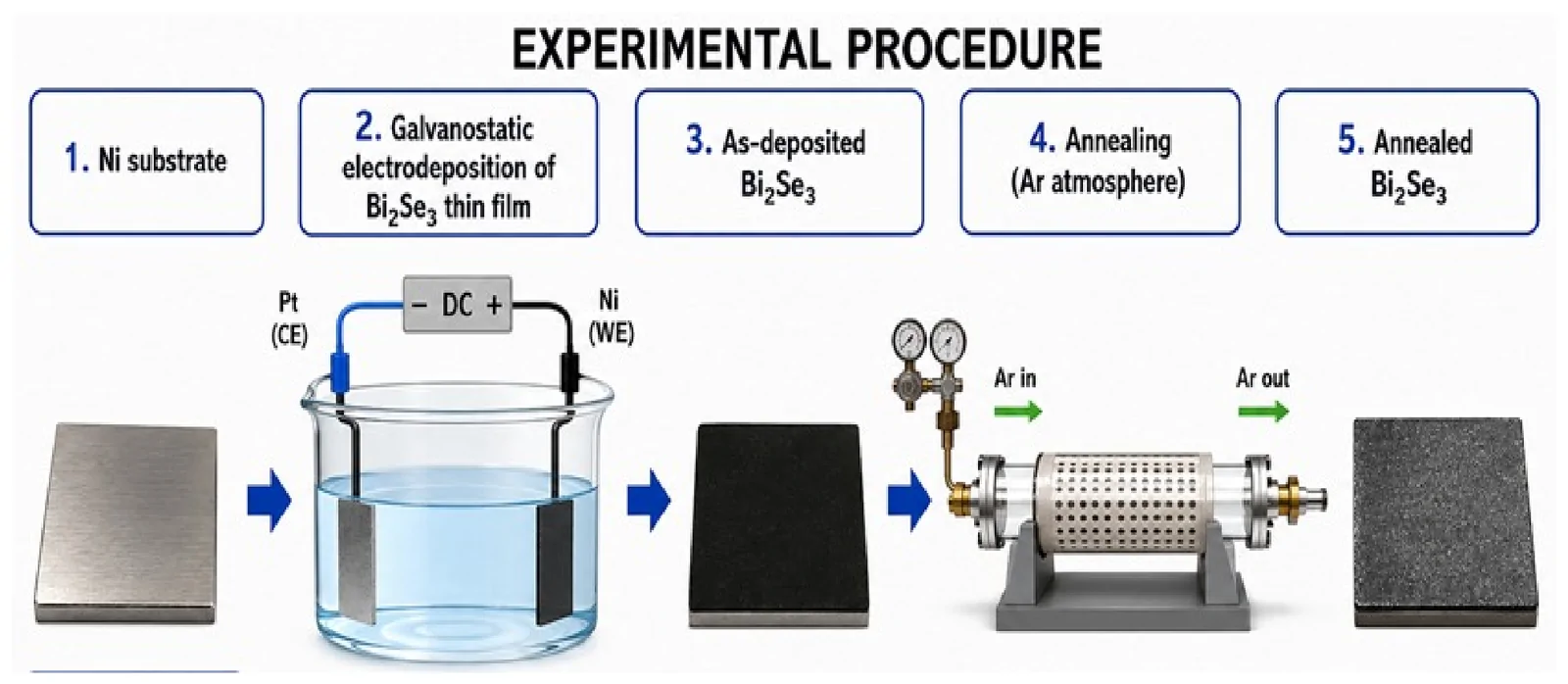
Schematic illustration of the preparation procedure of Ni/Bi_2_Se_3_ thin films by galvanostatic electrodeposition and subsequent annealing in an Ar atmosphere.

Bi_2_Se_3_ thin films were deposited galvanostatically onto Ni substrates from an ethylene glycol-based electrolyte containing 0.07 M Bi(NO_3_)_3_·5H_2_O (Sigma-Aldrich, Louis, MO, USA) and0.03 M H_2_SeO_3_ (Merck KGaA, Darmstadt, Germany), and 0.05 M citric acid (C_6_H_8_O_7_) (Sigma-Aldrich, Louis, MO, USA) as a complexing agent. The electro-deposition was carried out at room temperature using a current density of 0.05 mA cm^−2^ for 3 h. Ni substrates (2 cm^2^, thickness 0.1 mm) were used for film preparation and semiconductor characterization, whereas Pt electrodes were employed only in the electrochemical studies conducted to determine the deposition behavior and deposition potential region of Bi_2_Se_3_ formation. Following electrodeposition, the films were thermally annealed at 673 K (400 °C) for 1 h in an Ar atmosphere to improve crystallinity and phase purity (Figure 1). To ensure re-producibility, the electrodeposition experiments were repeated under identical conditions, and comparable structural and electrochemical results were obtained.

During the electrochemical deposition process, Bi_2_Se_3_ powder was obtained by gradually increasing the applied current density using a TH 6212 DC Power Supply. The resulting precipitate was collected by filtration using filter paper and subsequently washed with distilled water. This washing step was necessary because the deposition medium was an ethylene glycol-based solution, which has an oily nature; therefore, dilution and removal of residual organic species were carried out using water. After washing, the obtained powder was dried and then subjected to thermal treatment at 400 °C (673 K) to ensure phase formation and structural stabilization.

The morphology, surface topography, and elemental composition of the deposited Bi_2_Se_3_ films were examined using a scanning electron microscope (Sigma, Carl Zeiss, Germany). The phase composition and crystal structure were analyzed by X-ray diffraction using a D2 Phaser diffractometer (Bruker, Karlsruhe, Germany).

The thickness of the Bi_2_Se_3_ films was determined using a microinterferometer (MII-4, LOMO, St. Petersburg, Russia). Raman spectroscopy of the samples was performed using a Laser Raman Microspectroscopy System Nanofinder 30 (Tokyo Instruments, Tokyo, Japan) with an excitation wavelength of 532 nm.

The unknown functional groups, bond strength, and atomic masses of the substance present in the chemical compound Bi_2_Se_3_, thermally treated at 400 °C (673 K) both in thin film and powder, were analyzed by their spectra from an IR-Affinity-1FTIR spectrometer with a wavelength range between 4000 and 400 cm^−1^.

The optical measurements were carried out using an MDR-23 spectrometer (LOMO, St. Petersburg, Russia). The electrical measurements were performed using a 34465A digital multimeter (Keysight Technologies, CA, USA).

To measure the temperature dependence of the intrinsic electrical resistance of ${Bi}_{2}{Se}_{3}$ thin films, the films were also deposited onto Ni electrodes via the galvanostatic method. The temperature dependence of electrical conductivity was measured in the temperature range of 300–458 K.

After confirming the formation of the Bi_2_Se_3_ compound, its electrophysical, photoconductive, and photoelectrochemical properties were investigated using various analytical methods.

## 3. Results and Discussion

### 3.1. XRD and SEM Analysis

**Figure 2:**
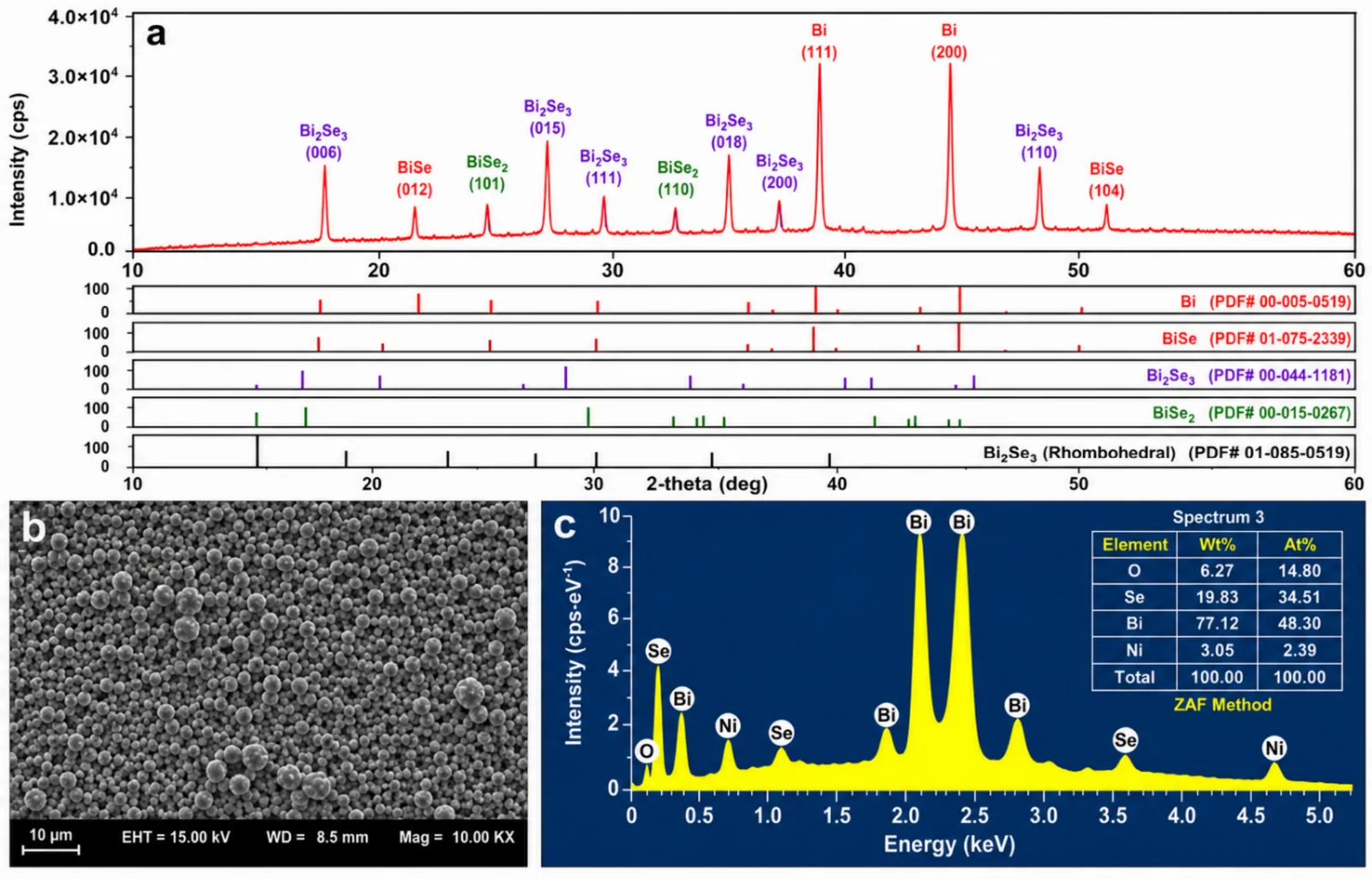
X-ray diffraction pattern (**a**), surface morphology (**b**), and elemental composition (**c**) of the as-deposited Bi_2_Se_3_ thin film electrodeposited on a Ni substrate before thermal treatment.

**Figure 3:**
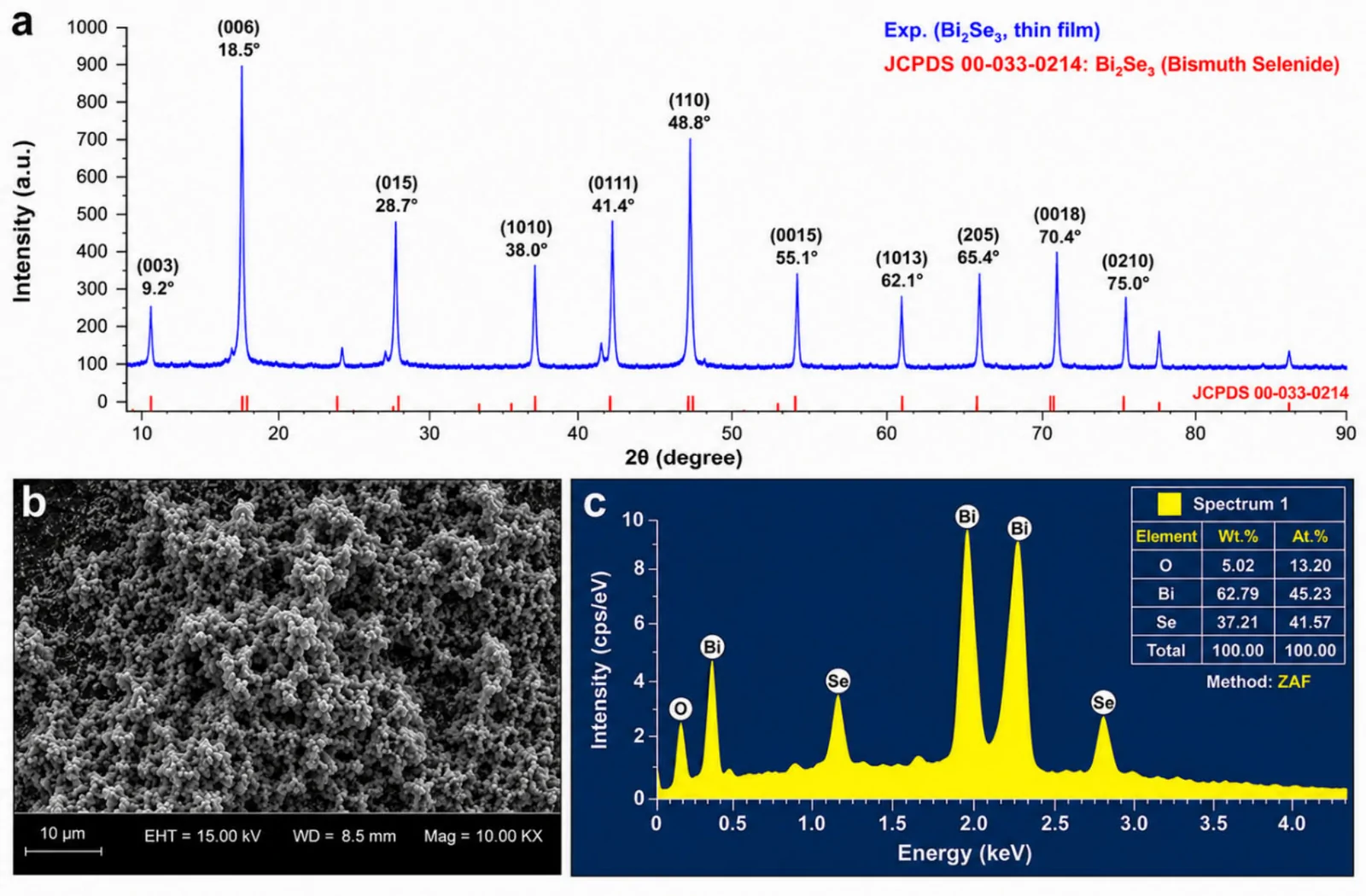
(**a**) XRD, (**b**) SEM, and (**c**) EDS analyses of the Bi_2_Se_3_ thin film electrodeposited on a Ni substrate after thermal treatment at 673 K in an Ar atmosphere.

To evaluate the effect of thermal treatment on the structural properties of the Bi_2_Se_3_ films deposited on Ni substrates, the XRD, SEM, and EDS patterns obtained before and after annealing were compared (Figure 2 and Figure 3) [6,7]. The XRD pattern of the as-deposited film (Figure 2a) confirms the formation of bismuth selenide phases on the Ni substrate during electrodeposition. The diffraction peaks observed at approximately 2θ = 18°, 29°, 38°, and 58° can be assigned to the rhombohedral Bi_2_Se_3_ phase. In addition to Bi_2_Se_3_, several weak reflections corresponding to BiSe, BiSe_2_, and elemental Bi are also detected, indicating that the electrodeposited film before thermal treatment is not completely phase-pure. The strong diffraction peaks located at 2θ ≈ 44.5° and 51.8° originate from the Ni substrate. The coexistence of secondary phases suggests incomplete crystallization and non-equilibrium phase formation during the electrodeposition process.

The surface morphology of the as-deposited Bi_2_Se_3_ film (Figure 2b) reveals a heterogeneous structure composed of irregularly shaped and agglomerated grains distributed non-uniformly across the substrate surface. The grain size varies approximately from 1 to 5 μm, indicating heterogeneous nucleation and growth during electrodeposition. The rough surface morphology and agglomerated grain structure are consistent with the XRD results, which indicate the coexistence of Bi_2_Se_3_ together with secondary phases such as BiSe, BiSe_2_, and elemental Bi.

The EDS spectrum (Figure 2c) confirms the presence of Bi and Se as the major constituents of the deposited layer, demonstrating successful co-deposition of both elements and the formation of a Bi–Se compound. The elemental composition is in good agreement with the XRD results and further verifies the successful formation of bismuth selenide on the Ni substrate. To improve crystallinity and phase purity, the as-deposited films underwent thermal treatment at 673 K (400 °C) for 1 h in an Ar atmosphere. Following thermal treatment, the formation of crystalline Bi_2_Se_3_ thin films on the Ni substrate was confirmed by XRD, SEM, EDS, and Raman analyses.

As shown in Figure 3a, the diffraction peaks located at 2θ values of approximately 9.2°, 18.5°, 28.7°, 38.0°, 41.4°, 48.8°, 55.1°, 62.1°, 65.4°, 70.4°, and 75.0° can be indexed to the rhombohedral Bi_2_Se_3_ phase (space group R3̅m, JCPDS No. 00-033-0214) [7,62]. Compared with the as-deposited film, the diffraction peaks become significantly sharper and more intense after thermal treatment, while reflections associated with secondary phases largely disappear. This behavior indicates improved crystallinity, enhanced phase purity, and reduced structural defects, demonstrating that thermal treatment promotes transformation toward the thermodynamically stable rhombohedral Bi_2_Se_3_ phase.

The SEM image (Figure 3b) reveals a compact granular morphology composed of closely packed crystallites, suggesting crystal growth and particle coalescence during thermal treatment. This microstructural evolution is consistent with the enhanced crystallinity observed in the XRD patterns. Furthermore, the EDS spectrum (Figure 3c) confirms that Bi and Se remain the dominant constituents of the film, with an atomic ratio close to the stoichiometric composition of Bi_2_Se_3_. A weak oxygen signal is also detected, which can be attributed to slight surface oxidation caused by exposure to ambient air during sample handling. This is likely due to the fact that argon purging in the setup leaves a small amount of oxygen.

Overall, the combined XRD, SEM, and EDS analyses indicate that Bi_2_Se_3_ formation occurs during electrodeposition; however, the presence of secondary phases and morphological inhomogeneity suggests that the as-deposited films possess limited crystallinity and incomplete phase development. Therefore, post-deposition thermal treatment was applied to promote phase transformation toward the thermodynamically stable Bi_2_Se_3_ phase and improve the structural quality of the films.

### 3.2. FTIR and Raman Spectroscopic Analysis

**Figure 4:**
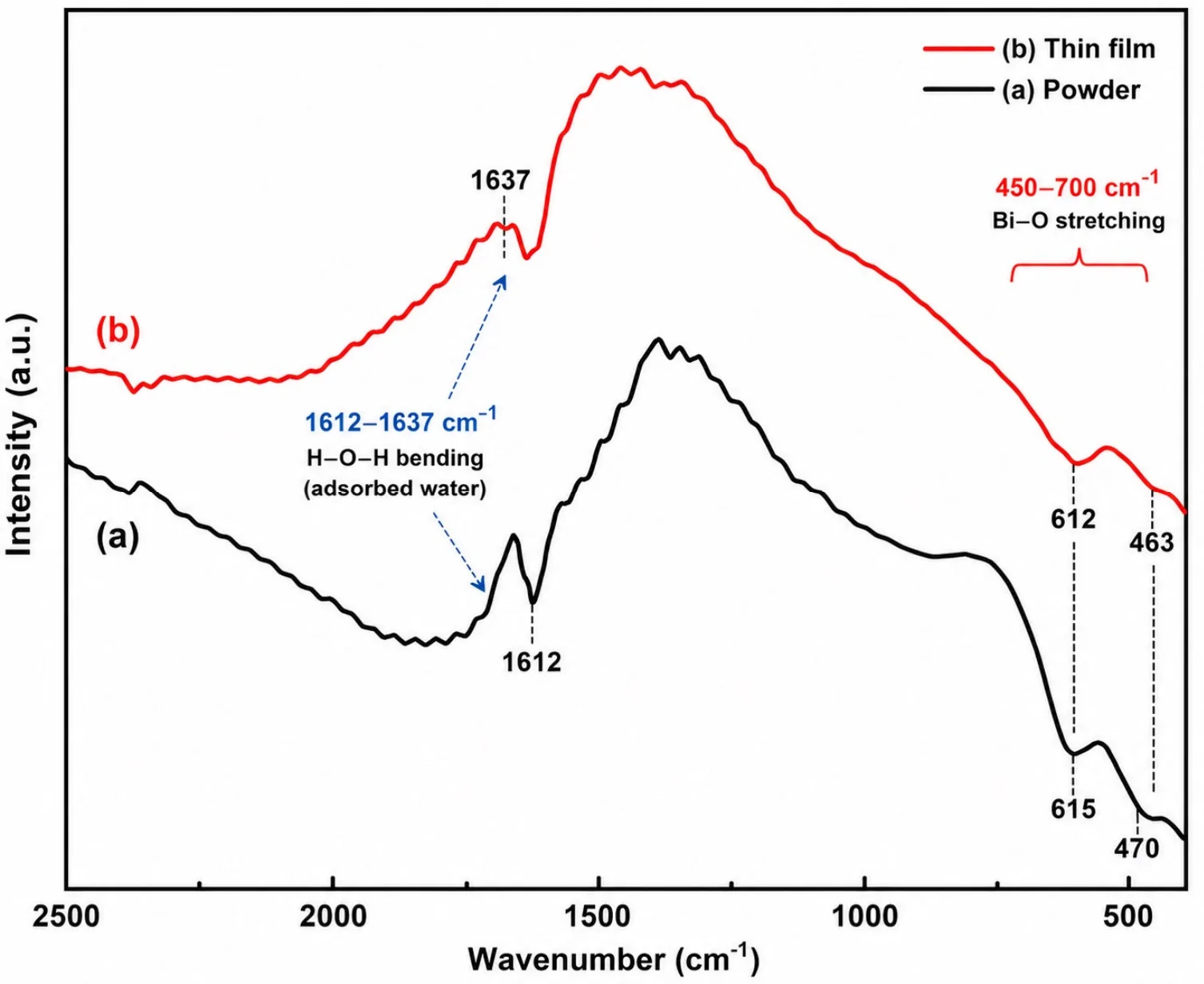
Comparative FTIR spectra of Bi_2_Se_3_ powder (pellet form) and electrodeposited thin film, showing characteristic absorption bands and Bi–O stretching vibrations in the 450–700 cm^−1^ region.

Figure 4 presents the FTIR spectra of Bi_2_Se_3_ in powder (a) and thin film (b) forms. Both spectra exhibit similar peak positions, confirming comparable chemical environments in the two forms. The absorption bands in the range of 450–700 cm^−1^ are attributed to Bi–O stretching vibrations, indicating that slight surface oxidation [63] has been intentionally included to support the assignment of Bi–O vibrational modes observed in the FTIR and Raman spectra. Although the primary focus of this study is Bi_2_Se_3_, the presence of minor surface oxidation (Bi_2_O_3_) necessitates the use of the relevant literature for proper interpretation of these features.

Figure 4 presents the FTIR spectra of Bi_2_Se_3_ powder (a) and the electrodeposited thin film (b). Both spectra exhibit similar absorption features, indicating that the chemical structure of the electrodeposited film closely resembles that of the powder precursor. Weak absorption bands observed in the 450–700 cm^−1^ region are attributed to Bi–O stretching vibrations, suggesting slight surface oxidation of Bi_2_Se_3_ during heat treatment [61]. In addition, the bands located at approximately 1612–1637 cm^−1^ are assigned to H–O–H bending vibrations of adsorbed water molecules and surface hydroxyl groups [63,64]. Similar oxidation-related features have been reported for Bi_2_Se_3_ synthesized by various methods and are generally associated with exposure of the material surface to ambient air. The relatively low intensity of the Bi–O bands indicates that oxidation is limited to the near-surface region and does not significantly affect the overall phase composition of the films.

**Figure 5:**
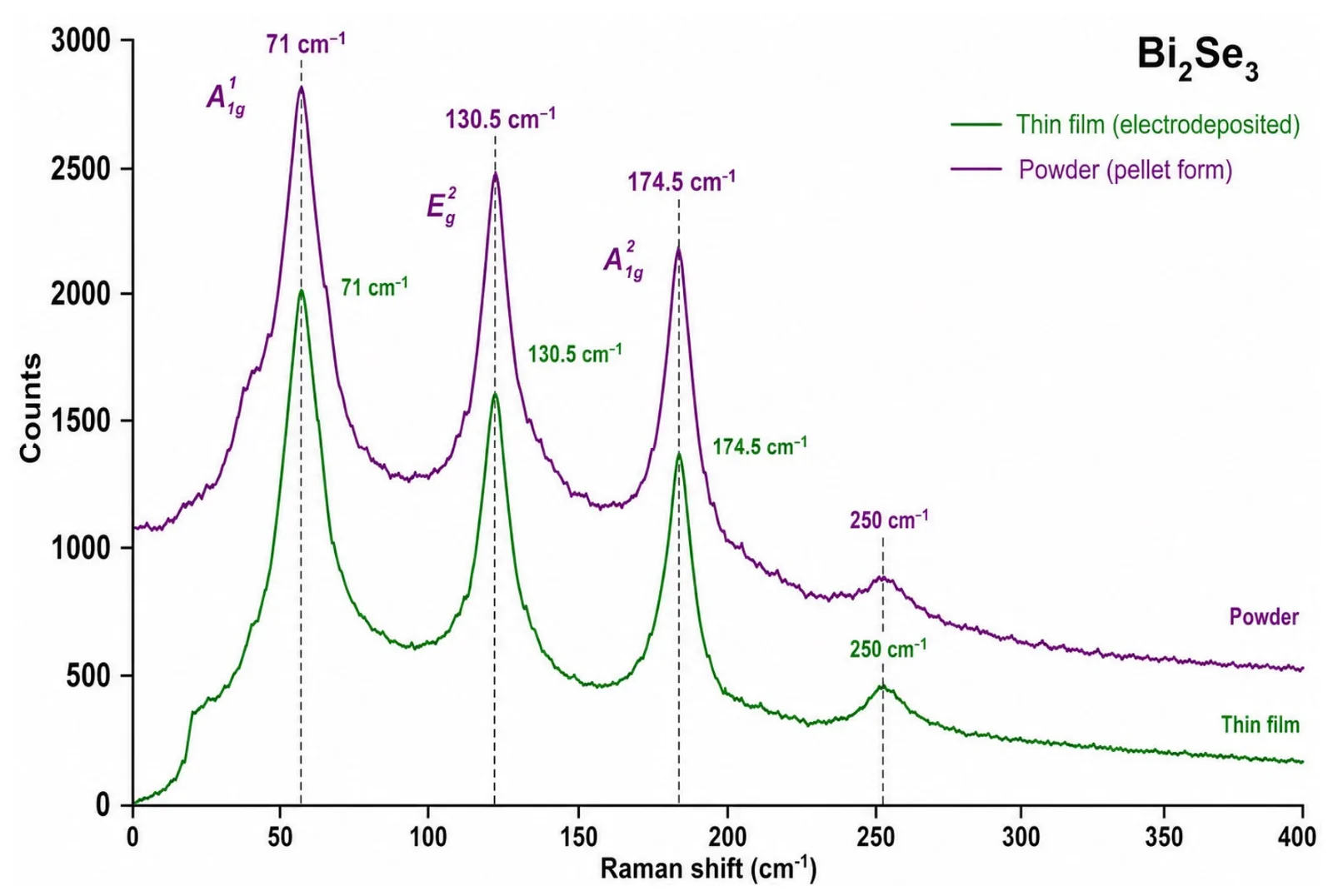
Comparative Raman spectra of Bi_2_Se_3_ powder (pellet form) and electrodeposited thin film, showing the characteristic A_1_g^1^, E_g^2^, and A_1_g^2^ vibrational modes of rhombohedral Bi_2_Se_3_.

According to the literature, the vibrational modes of Bi_2_Se_3_ lie within the wavenumber range of approximately 70–300 cm^−1^; therefore, low-wavenumber Raman spectroscopy was employed in the investigations (Figure 5) [14,65,66,67].

The Raman spectra of the Bi_2_Se_3_ powder and electrodeposited thin film are shown in Figure 5. Both spectra exhibit the characteristic Raman-active vibrational modes of rhombohedral Bi_2_Se_3_, confirming successful formation and preservation of the crystal structure after electrodeposition. The peaks located at approximately 71, 130.5, and 174.5 cm^−1^ are assigned to the A_1_g^1^, Eg^2^, and A_1_g^2^ modes, respectively, and are in good agreement with previously reported values for crystalline Bi_2_Se_3_. The presence of these well-defined vibrational modes confirms the layered rhombohedral structure and good crystallinity of both samples [68,69,70,71].

A comparison of the Raman spectra reveals that the thin film exhibits peak positions nearly identical to those of the powder sample, indicating that the electrodeposition process preserves the fundamental crystal structure of Bi_2_Se_3_. Minor differences in peak intensity and band broadening can be attributed to variations in crystallite size, preferred orientation, surface morphology, and defect concentration between the powder and thin-film forms. In addition, a weak broad feature around 250 cm^−1^ is observed in both spectra, which may be associated with second-order Raman scattering or a small degree of structural disorder within the Bi_2_Se_3_ lattice.

The FTIR and Raman results are fully consistent with the XRD analysis. While FTIR spectroscopy reveals weak Bi–O vibrations associated with slight surface oxidation, no Raman bands corresponding to crystalline Bi_2_O_3_ are detected. Together with the XRD results, this indicates that Bi_2_Se_3_ remains the dominant crystalline phase and that oxidation is confined to a thin surface layer. Therefore, the combined spectroscopic analyses confirm that the electrodeposition process successfully produces crystalline Bi_2_Se_3_ thin films with structural characteristics closely resembling those of the original powder material.

### 3.3. Semiconductor Properties

It is well known that semiconductor deposits often exhibit complex heterogeneous morphologies during electrodeposition, attracting considerable interest because of their unique structural and electrical properties [72].

To investigate the electrical properties of the obtained Bi_2_Se_3_ material and the interaction between the deposited semiconductor and the substrate, current–voltage characteristics (CVCs) were measured using both electrodeposited Bi_2_Se_3_ films on Ni substrates and Bi_2_Se_3_ samples in pellet form. The use of these two sample configurations enabled a comparative evaluation of charge transport behavior in the thin-film and bulk forms of the material. To assess the homogeneity and reproducibility of the electrical response, the measurements were performed at several different locations on the sample surface.

**Figure 6:**
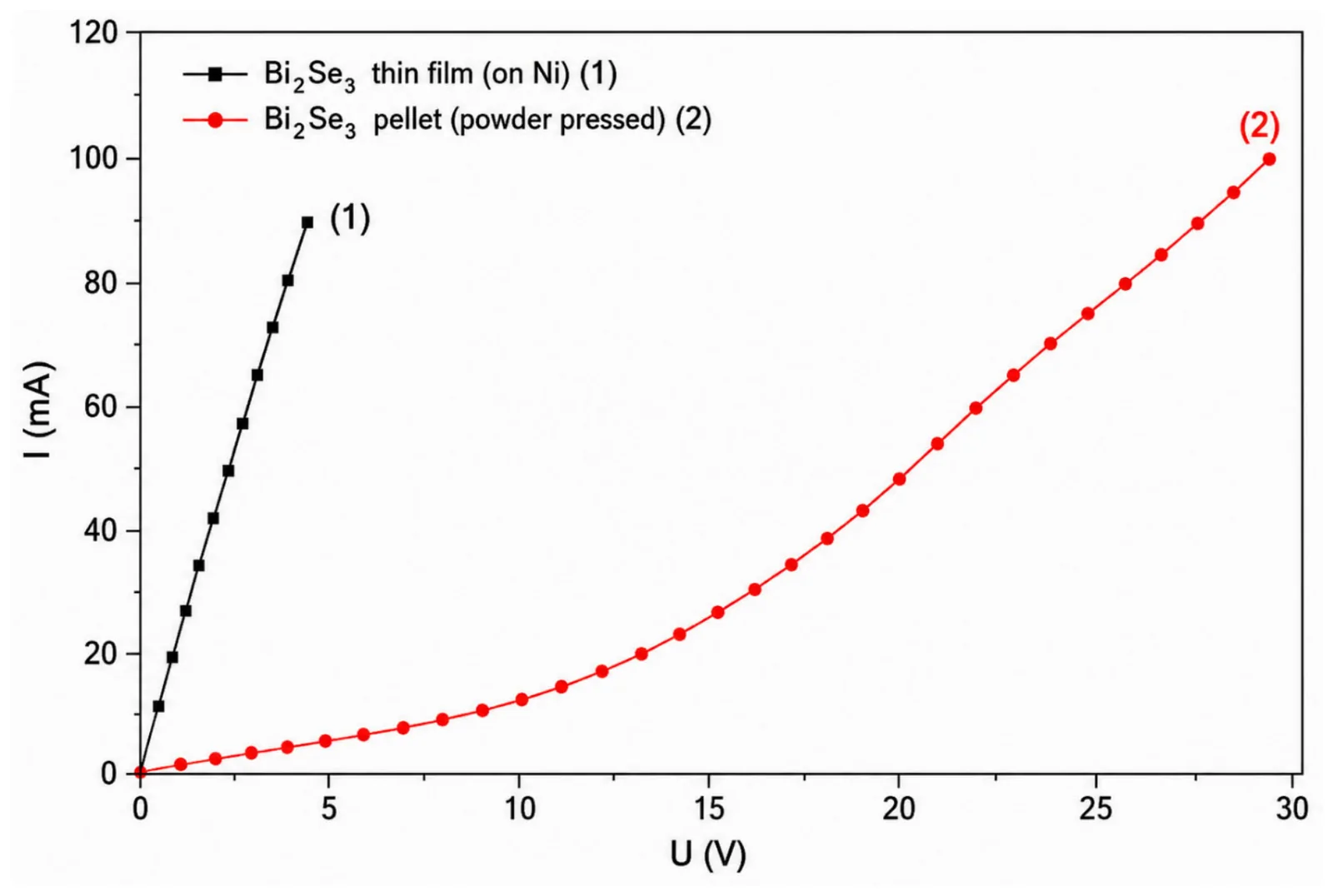
Comparative current–voltage (I–V) characteristics of (1) electrodeposited Bi_2_Se_3_ thin film on Ni substrate and (2) Bi_2_Se_3_ pellet prepared from pressed powder.

Figure 6 compares the current–voltage (I–V) characteristics of the electrodeposited Bi_2_Se_3_ thin film deposited on a Ni substrate and the Bi_2_Se_3_ pellet prepared from pressed powder. A significant difference in electrical behavior is observed between the two sample configurations.

The Bi_2_Se_3_ thin film exhibits an approximately linear dependence of current on the applied voltage, indicating an ohmic-like electrical response. This behavior is mainly associated with the highly conductive Ni substrate, which facilitates charge transport and reduces the overall resistance of the film/substrate structure. The good electrical contact between the Bi_2_Se_3_ layer and the metallic substrate further contributes to the nearly linear current–voltage dependence.

In contrast, the Bi_2_Se_3_ pellet exhibits a pronounced nonlinear current–voltage characteristic, which is typical of semiconducting materials. Since the pellet was prepared from pressed Bi_2_Se_3_ powder without a metallic substrate, the measured response predominantly reflects the bulk electrical properties of Bi_2_Se_3_. The nonlinear increase in current with increasing voltage suggests the involvement of field-dependent carrier transport and grain-boundary effects commonly observed in polycrystalline semiconducting materials.

The comparison between the thin-film and pellet samples demonstrates the strong influence of substrate conductivity, contact resistance, and sample architecture on the electrical transport properties of Bi_2_Se_3_. While the thin-film structure exhibits an almost ohmic response due to the presence of the conductive Ni substrate, the pellet more clearly reveals the intrinsic semiconducting behavior of Bi_2_Se_3_. These results confirm that the electrical characteristics of Bi_2_Se_3_ are strongly dependent on sample configuration and microstructure, which should be considered in the design of electronic and sensing devices.

**Figure 7:**
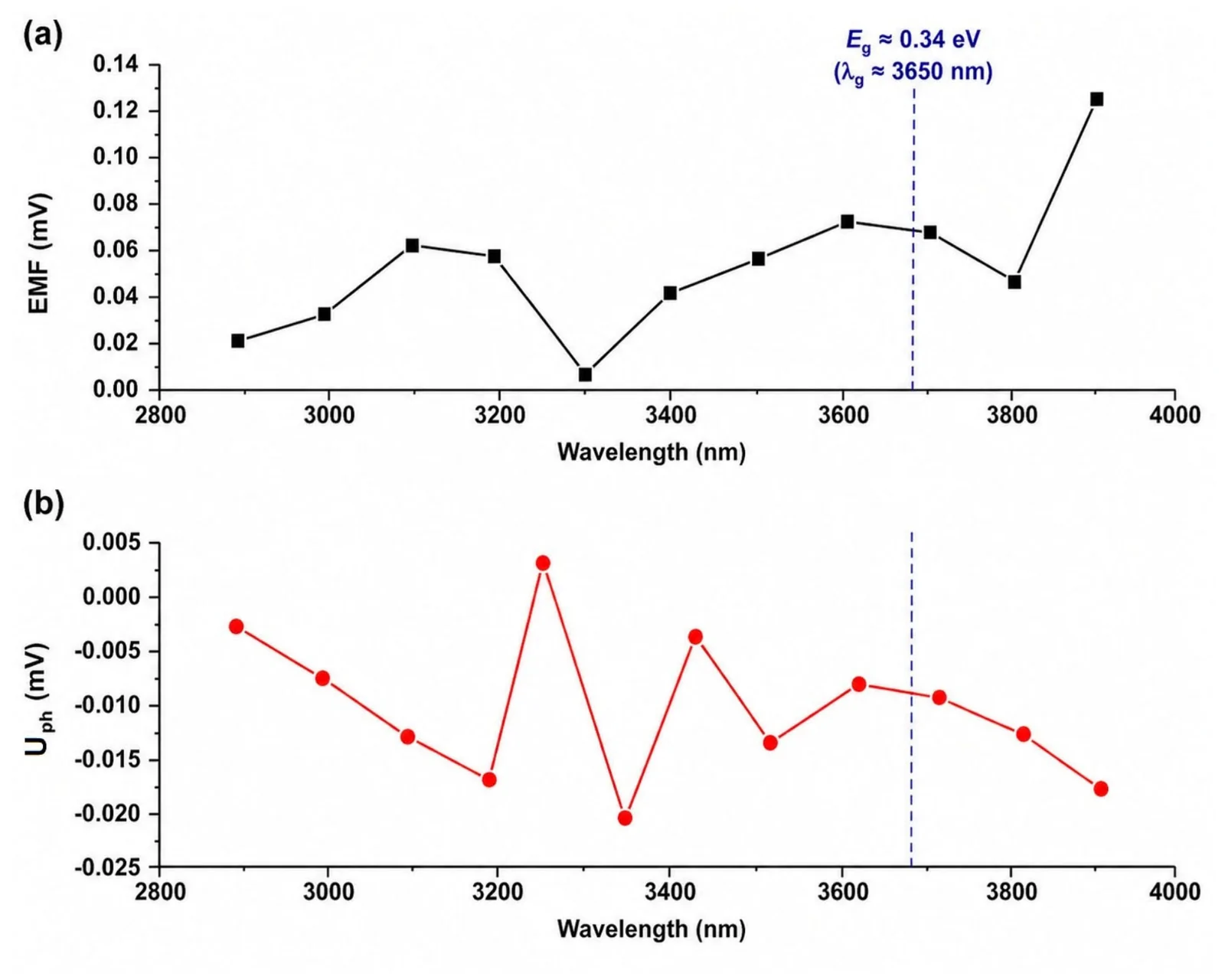
Spectral EMF (**a**) and mid-infrared photoconductivity (**b**) responses of electrodeposited Bi_2_Se_3_ thin films on a Ni substrate as a function of wavelength.

To further investigate the photoelectrical properties of the electrodeposited Bi_2_Se_3_ thin films, the spectral dependence of the electromotive force (EMF) was studied using the capacitor method under modulated infrared illumination (Figure 7). The spectral EMF response provides information about the interaction of electromagnetic radiation with the semiconductor, including photoexcitation processes, charge-carrier generation, and energy transitions associated with the electronic structure of the material. Analysis of the wavelength-dependent EMF response can therefore be used to evaluate the spectral sensitivity of the films and to identify photoresponse features occurring near the band-gap energy of Bi_2_Se_3_.

The spectral dependences of the electromotive force (EMF) and photoconductivity of the electrodeposited Bi_2_Se_3_ thin films were investigated in the wavelength range of 2800–4000 nm under modulated mid-infrared (mid-IR) illumination (Figure 7). The EMF and photoconductivity spectra exhibit a measurable photoresponse throughout the investigated spectral range, confirming the generation of nonequilibrium charge carriers under infrared irradiation.

The EMF spectrum (Figure 7a) shows several local maxima and minima, indicating that the photoresponse is influenced by carrier generation, trapping, and recombination processes. A similar non-monotonic behavior is observed in the photoconductivity spectrum (Figure 7b), suggesting the participation of multiple electronic states in the photoexcitation process.

Particular attention should be paid to the spectral region around 3400–3900 nm, corresponding to photon energies of approximately 0.32–0.36 eV. These values are close to the reported band-gap energy of Bi_2_Se_3_ (Eg ≈ 0.34 eV). Therefore, the enhanced photoresponse observed in this wavelength range can be attributed to photoexcitation processes occurring near the band-edge energy of the material.

The observed photoconductivity in the 2800–4000 nm spectral region is primarily related to the narrow-band-gap nature of Bi_2_Se_3_, which enables the absorption of infrared radiation and the generation of charge carriers. However, the measured photoresponse is also influenced by the Ni/Bi_2_Se_3_ interface. Due to the difference in work functions between Ni and Bi_2_Se_3_, a Schottky-type barrier may form at the metal–semiconductor junction, creating an internal electric field that promotes the separation and transport of photogenerated carriers. Therefore, the infrared photoresponse cannot be attributed solely to either the bulk Bi_2_Se_3_ film or the interface. Instead, it is likely the result of the combined contribution of intrinsic carrier generation in Bi_2_Se_3_ and interface-assisted carrier separation at the Ni/Bi_2_Se_3_ contact. While direct characterization of the barrier height was not performed in the present work, the nonlinear current–voltage behavior and the appearance of photoinduced voltage support the existence of an interface-assisted contribution to the observed infrared response.

The obtained results demonstrate that the electrodeposited Bi_2_Se_3_ thin films are sensitive to mid-infrared radiation and exhibit photoelectrical activity in the 2.8–4.0 μm spectral region. Such characteristics indicate the potential applicability of these films in infrared-sensitive optoelectronic and sensing devices.

**Figure 8:**
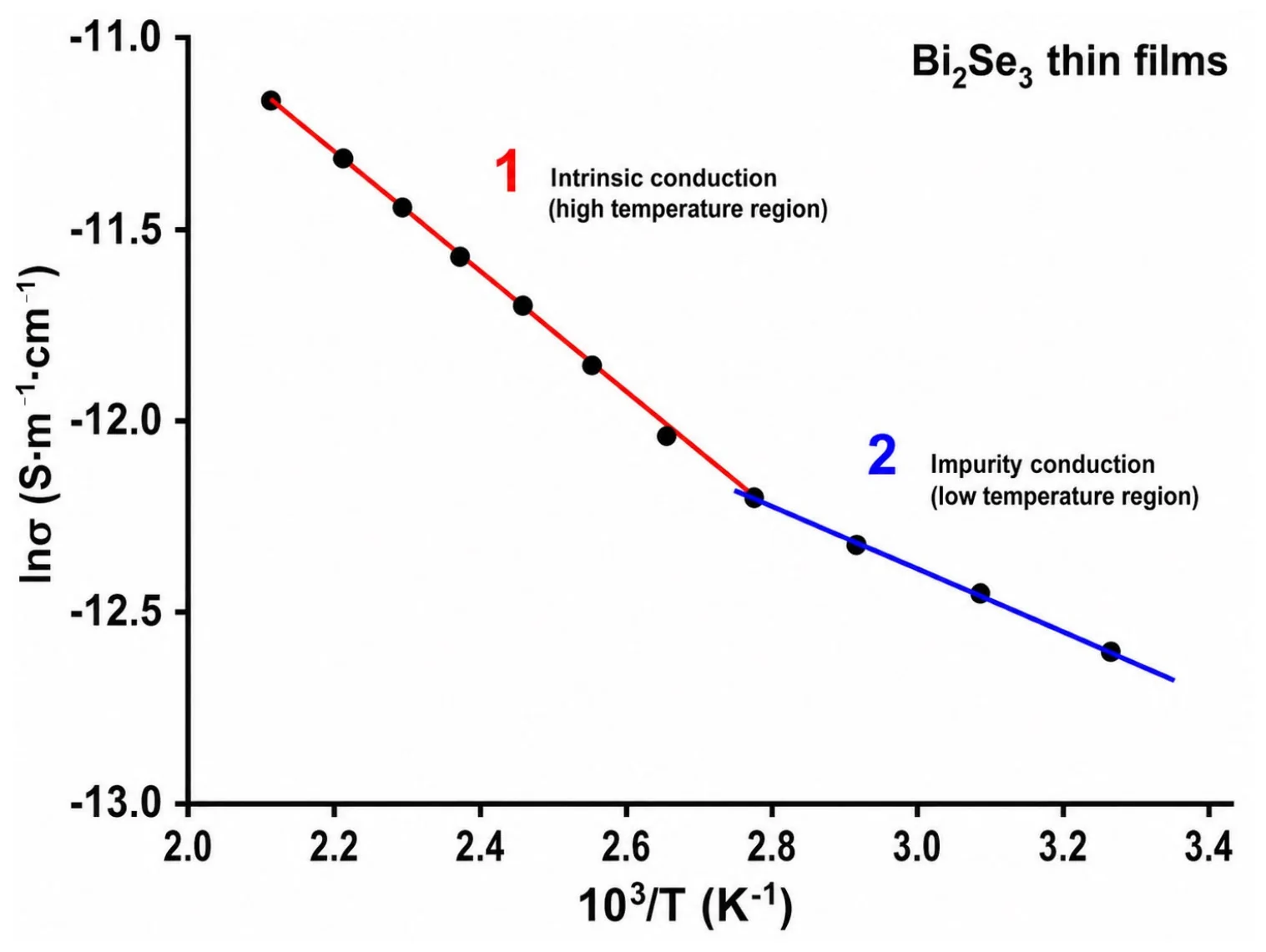
Arrhenius plot (lnσ versus 10^3^/T) of the conductivity of electrodeposited Bi_2_Se_3_ thin films, revealing two conduction regimes associated with impurity-assisted and intrinsic charge transport.

To investigate the temperature dependence of the conductivity of the electrodeposited Bi_2_Se_3_ thin films, the resistance of the prepared samples was measured in the temperature range of 300–458 K. The conductivity data were analyzed using the Arrhenius relation by plotting lnσ as a function of 10^3^/T (Figure 8).

As shown in Figure 8, two linear regions with different slopes can be distinguished in the temperature dependence of the electrical conductivity. Such behavior is characteristic of semiconductors containing impurity and defect states. The low-temperature region (300–353 K) is associated with impurity-assisted conduction, where charge transport is predominantly governed by carriers originating from impurity and defect levels. In contrast, at temperatures above 353 K, the concentration of thermally generated charge carriers increases significantly, resulting in a gradual transition to intrinsic conduction.

The activation energies calculated from the slopes of the corresponding linear regions were found to be 0.08 eV for impurity conduction and 0.25 eV for intrinsic conduction. Based on the obtained activation energy, the temperature sensitivity coefficient was determined to be B = 2903 K. The temperature coefficients of resistance were calculated as α^300^ = 0.032 K^−1^ and α^458^ = 0.013 K^−1^.

Based on the activation energy obtained for the intrinsic conduction region, the temperature sensitivity coefficient (B) was calculated using the relation B = Ea/k, yielding a value of 2903 K. The temperature coefficient of electrical resistivity (α) was subsequently determined from the expression α = B/T^2^. The calculated values of Ea, B, and α are summarized in Table 1.

It should be noted that the activation energy obtained from temperature-dependent conductivity measurements differs from the optical band-gap energy reported for Bi_2_Se_3_. The activation energies describe thermally activated carrier transport through intrinsic and defect-related states, whereas the optical band gap is associated with electronic transitions between the valence and conduction bands. Therefore, the obtained activation energy values should not be interpreted as direct measurements of the optical band gap.

### 3.4. Chronoamperometry

To gain further insight into the electrodeposition mechanism of Bi_2_Se_3_, chronoamperometric (CA) measurements were performed at selected deposition potentials determined from the cyclic voltammograms. Analysis of the current–time transients allows the nucleation and growth behavior of the deposited films to be evaluated.

**Figure 9:**
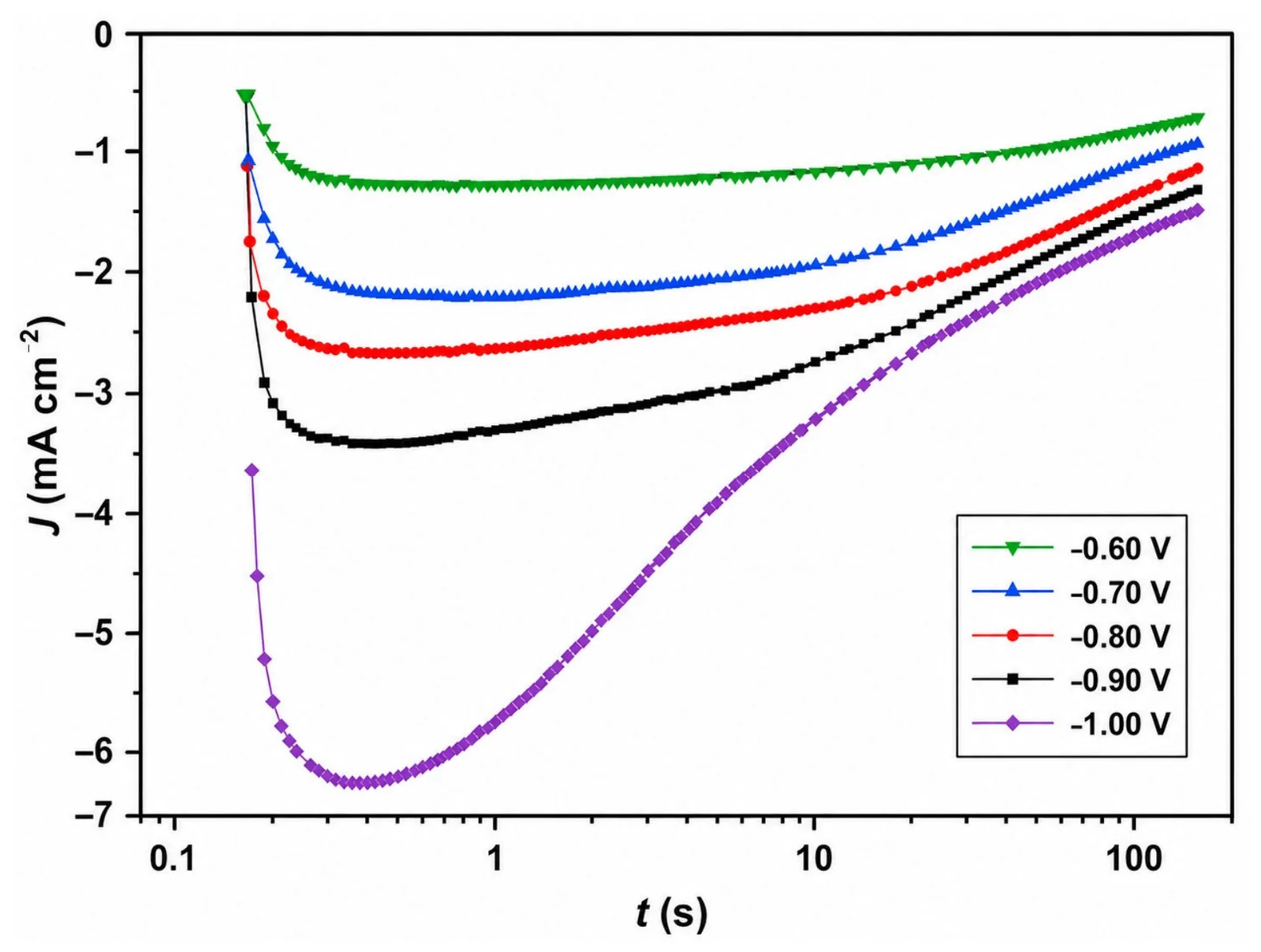
Current–time curves for the electrodeposition of Bi_2_Se_3_ on a Ni electrode from an electrolyte containing 0.07 M Bi(NO_3_)_3_·5H_2_O, 0.03 M H_2_SeO_3_, and citric acid (C_6_H_8_O_7_) at various deposition potentials: 1—(−0.60), 2—(−0.70), 3—(−0.80), 4—(−0.90), and 5—(−1.00) V vs. Ag/AgCl.

Current–time (I–t) transients were recorded at applied potentials of −0.6, −0.7, −0.8, −0.9, and −1.0 V at room temperature, as shown in Figure 9. Immediately after the application of the deposition potential, the current decreases sharply due to the charging of the electrical double layer at the electrode/electrolyte interface. This initial stage is followed by an increase in current associated with the nucleation and growth of the deposited Bi_2_Se_3_ phase, resulting in an expansion of the electroactive surface area.

**Figure 10:**
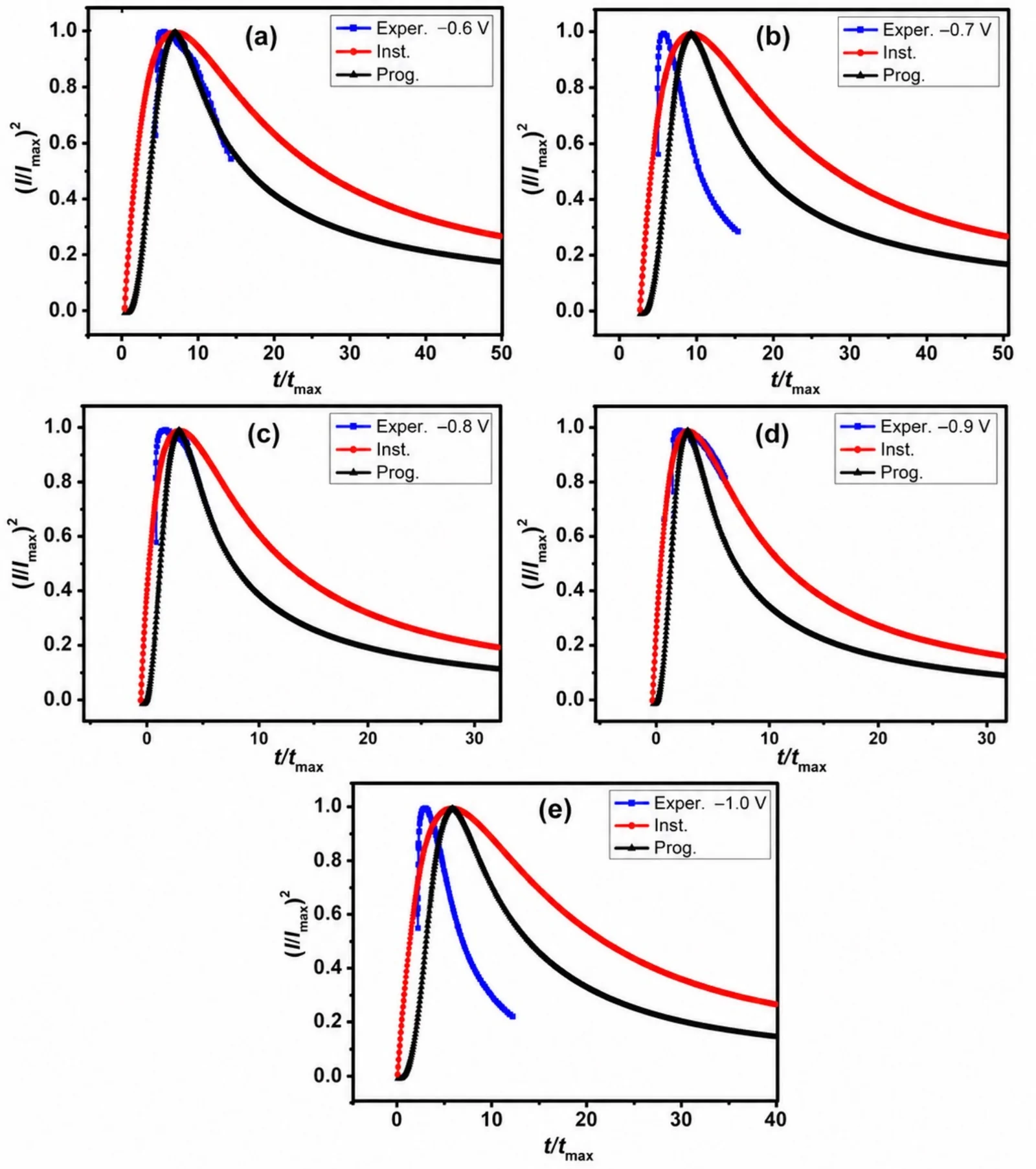
Dimensionless plots of (*I*/*I_max_*)^2^ versus (*t*/*t_max_*) obtained from chronoamperometric transients recorded during Bi_2_Se_3_ electrodeposition. The experimental data (blue curves) are compared with the theoretical Scharifker–Hills transients for instantaneous (red curves) and progressive (black curves) three-dimensional nucleation. Electrodeposition was carried out from an electrolyte containing 0.07 M Bi(NO_3_)_3_·5H_2_O, 0.03 M H_2_SeO_3_, and citric acid in ethylene glycol at different deposition potentials: (**a**) −0.6 V, (**b**) −0.7 V, (**c**) −0.8 V, (**d**) −0.9 V, and (**e**) −1.0 V.

As the deposition potential becomes more negative, the measured current density increases, reflecting enhanced electrochemical reduction and film growth rates. The nucleation and growth behavior of Bi_2_Se_3_ was analyzed using the Scharifker–Hills (S–H) model [73] by comparing the experimental current transients with the theoretical curves for three-dimensional instantaneous and progressive nucleation under diffusion-controlled conditions, as shown in Figure 10a–d. The corresponding theoretical relationships are given by Equations (1) and (2), respectively.

(1) It2/Imax=1.9542/ttmax 1−exp−1.2564 t/tmax2

(2) It2/Imax=1.2254/ttmax 1−exp−2.3367 t/tmax22

Figure 10a–e present the dimensionless plots of (*I*/*I_max_*)^2^ versus (*t*/*t_max_*) obtained from the chronoamperometric transients shown in Figure 9. The experimental data were compared with the theoretical Scharifker–Hills transients corresponding to instantaneous and progressive three-dimensional nucleation.

The solid black and red lines represent the theoretical transient processes for instantaneous and progressive nucleation, respectively, as shown in Figure 10, while the blue lines represent the experimental data. The nucleation and growth processes of Bi_2_Se_3_ under these conditions can be accurately predicted from Figure 10a–d. Initially, the experimental data align well with the progressive nucleation model, according to which bismuth nucleation occurs at multiple electrochemically active centers on the surface of the platinum electrode. Subsequently, the electrodeposition process deviates from the Sharifker–Hills instantaneous nucleation model, as illustrated in Figure 10a–d. This deviation can be attributed to a transition in the growth mechanism, where nucleation is followed by crystal growth predominantly governed by diffusion-controlled kinetics.

Over time, the nucleation and growth of Bi_2_Se_3_ during electrodeposition transition to a mixed-control regime governed by both diffusion and charge-transfer kinetics. The observed deviation from the ideal nucleation behavior may also be attributed to concurrent hydrogen evolution reactions occurring during the early stages of deposition, which can influence the local environment and alter the morphology of the resulting nuclei. Nevertheless, the experimental data presented in Figure 10d show strong agreement with the theoretical model of progressive three-dimensional nucleation and growth, particularly at a deposition potential of approximately −0.6 V.

Additional information about the growth mechanism can be obtained by calculating the nucleation active center density (N_0_) on the electrode surface under the below conditions.

As shown in Figure 10, the experimental transients exhibit only partial agreement with the theoretical models. The closest correspondence is observed in the vicinity of the current maximum, particularly at deposition potentials between −0.6 and −0.9 V, where the experimental curves approach the progressive nucleation model. However, noticeable deviations are observed at longer deposition times, indicating that the electrodeposition process cannot be fully described by the ideal assumptions of the Scharifker–Hills model.

The deviation between the experimental and theoretical curves suggests that additional processes contribute to Bi_2_Se_3_ film growth during electrodeposition. At more negative potentials, hydrogen evolution and charge-transfer limitations may influence the current response, resulting in departures from the ideal diffusion controlled nucleation behavior. Consequently, the deposition process gradually evolves from an initially nucleation-controlled stage toward a mixed-control regime involving both diffusion and charge-transfer kinetics.

Among the investigated conditions, the transient recorded at −0.6 V exhibits the closest resemblance to the theoretical progressive nucleation model, whereas larger deviations are observed at −0.7 and −1.0 V. These results indicate that Bi_2_Se_3_ electrodeposition proceeds through a complex nucleation and growth mechanism involving simultaneous nucleation, crystal growth, diffusion, and interfacial charge-transfer processes.

Additional information regarding the growth mechanism can be obtained by calculating the nucleation active-center density (*N*_0_) on the electrode surface under these conditions.

(3) $$N_{0}=0.065{\left(8\pi CM/\rho \right)}^{-1/2}{\left(zfC/t_{max}i_{max}\right)}^{2}$$

where *C* is the bulk concentration of the electroactive species (mol cm^−3^), *z* is the number of electrons involved in the electrodeposition process, *f* is the Faraday constant, *M* is the molar mass of the deposited material (g mol^−1^), and *ρ* is its density (g cm^−3^). The diffusion coefficient (D) of the electroactive species in the electrolyte can be estimated from chronoamperometric data. According to the Scharifker–Hills nucleation model, D is related to the maximum current density (*i_max_*) and the time required to reach the current maximum (*t_max_*) through the following relationship [73,74]:

(4) $$D\;=\;i_{max}^{2}t_{max}^{2}/0.1629(zfC)$$

The values of *i_max_*, *t_max_*, D, and *N*_0_ calculated at different deposition potentials are summarized in Table 2. As the deposition potential becomes more negative, variations in the *i_max_·t_max_* product are observed, reflecting changes in the nucleation and growth kinetics of the deposited Bi_2_Se_3_ phase. The obtained results suggest that the electrodeposition process is influenced by both mass transport and interfacial kinetic effects rather than by purely diffusion-controlled growth. The calculated diffusion coefficients are relatively low, which may be attributed to the high viscosity of the ethylene glycol-based electrolyte. In addition, the diffusion coefficient exhibits a dependence on the applied deposition potential, indicating that the transport of electroactive species and the electrodeposition kinetics are affected by the polarization conditions. The corresponding values are listed in Table 2.

The calculated diffusion coefficients (D) and nucleation site densities (N_0_) obtained from chronoamperometric analysis are summarized in Table 2. The diffusion coefficient varies within the range of 2.29 × 10^−7^–3.77 × 10^−7^ cm^2^ s^−1^, indicating that mass transport remains an important factor throughout the investigated potential range. In contrast, the calculated nucleation density (N_0_) does not exhibit a strictly monotonic dependence on the applied deposition potential but fluctuates across the investigated potential range. This behavior suggests that the density of active nucleation sites is governed not only by the applied overpotential but also by factors such as local surface conditions, surface heterogeneity, charge-transfer kinetics, and mass-transport effects. The non-monotonic variation in N_0_ is consistent with the deviations observed between the experimental and theoretical Scharifker–Hills transients (Figure 10), indicating that the electrodeposition process cannot be fully described by an ideal nucleation model. These deviations become more pronounced at higher cathodic overpotentials, where additional processes, including hydrogen evolution and interfacial charge-transfer limitations, may significantly influence nucleation and growth behavior. Furthermore, the relatively high viscosity of the ethylene glycol-based electrolyte contributes to slower mass transport of electroactive species, which can affect both the nucleation rate and subsequent crystal growth. Overall, the results suggest a transition from a predominantly nucleation-controlled process to a mixed diffusion/charge-transfer-controlled regime as the cathodic potential becomes more negative.

## 4. Conclusions

The structural, electrical, photoelectrical, and chronoamperometric properties of Bi_2_Se_3_ thin films electrodeposited from a non-aqueous ethylene glycol-based electrolyte were investigated. SEM, EDS, XRD, FTIR, and Raman analyses confirmed the formation of crystalline Bi_2_Se_3_ films with a thickness of 4–6 μm. Comparison of the as-deposited and annealed samples demonstrated that thermal treatment at 673 K significantly improved the crystallinity and phase purity of the films. Raman spectroscopy revealed the characteristic A_1_g^1^, E_g^2^, and A_1_g^2^ vibrational modes of rhombohedral Bi_2_Se_3_, while FTIR measurements indicated only minor surface oxidation.

The current–voltage characteristics and temperature-dependent electrical conductivity of the films were studied. Analysis of the Arrhenius plots revealed two conduction regimes associated with impurity-assisted and intrinsic charge transport. The activation energies were determined to be 0.08 eV and 0.25 eV for the impurity and intrinsic conduction regions, respectively. The temperature sensitivity coefficient was calculated as B = 2903 K, while the temperature coefficients of resistance were α^300^ = 0.032 K^−1^ and α^458^ = 0.013 K^−1^. Spectral EMF and photoconductivity measurements demonstrated the sensitivity of the Bi_2_Se_3_ films to mid-infrared radiation in the wavelength range of 2.8–4.0 μm, confirming their photoactive behavior. The enhanced photoresponse observed near 3.4–3.9 μm was attributed to photoexcitation processes occurring close to the band-edge energy of Bi_2_Se_3_. Chronoamperometric analysis based on the Scharifker–Hills model showed that the electrodeposition process proceeds through a complex nucleation and growth mechanism. The experimental transients exhibited partial agreement with the theoretical progressive nucleation model and indicated a gradual transition from nucleation-controlled growth to a mixed diffusion/charge-transfer-controlled regime. The calculated nucleation density and diffusion coefficient values further confirmed the influence of both mass transport and interfacial kinetic effects on film formation.

Overall, the obtained results demonstrate that electrodeposited Bi_2_Se_3_ thin films possess promising structural, electrical, and photoelectrical properties, making them attractive candidates for infrared-sensitive optoelectronic and photoelectrochemical applications.

## Figures and Tables

**Table 1:** Electrical parameters of electrodeposited Bi_2_Se_3_ thin films determined from temperature-dependent conductivity measurements.

E_a_^intrins^, eV	E_a_^doped^, eV	B, (K)	α^300^ (K^−1^)	α^458^ (K^−1^)
0.25	0.08	2903	0.032	0.013

**Table 2:** Experimental data from CA electrodeposition of Bi_2_Se_3_ on a Ni substrate.

E (V)	i_max_ (mA)	t_max_ (s)	D (cm^2^ s^−1^)	N_0_
−0.60	1.472	6.0	2.77 × 10^−7^	1.23 × 10^6^
−0.70	1.733	8.4	2.29 × 10^−7^	1.47 × 10^6^
−0.80	1.809	3.8	3.31 × 10^−7^	0.64 × 10^6^
−0.90	1.993	3.8	2.52 × 10^−7^	0.79 × 10^6^
−1.00	2.707	2.8	3.77 × 10^−7^	1.21 × 10^6^

## Data Availability

The original contributions presented in this study are included in the article. Further inquiries can be directed to the corresponding author.
